# Supplementary material for: A Review of Recent Advances in Computer-Aided Detection Methods Using Hyperspectral Imaging Engineering to Detect Skin Cancer
Source: Cancers (Basel). 2023 Nov 29;15(23):5634. doi: 10.3390/cancers15235634 (PMC10705122; doi:10.3390/cancers15235634)
Supplement: Supplementary file 1 [file cancers-15-05634-s001.zip › cancers-2588597-supplementary-updated-done.pdf]

cancers

MDPI

Review

A Systematic Meta-Analysis of Computer-Aided Detection Methods using Hyperspectral Imaging Engineering to Detect Skin Cancer: Supplement Material

Hung-Yi Huang [1], Yu-Ping Hsiao [2,3], Riya Karmakar [4], Arvind Mukundan [4], Pramod Chaudhary [5], Shang-Chin Hsieh [6,*] and Hsiang-Chen Wang [4,7,8] *

1 Department of Dermatology, Ditmanson Medical Foundation Chiayi Christian Hospital, Chia Yi City 60002, Taiwan. huanghungyi1@gmail.com (H.Y.H.)
2 Department of Dermatology, Chung Shan Medical University Hospital, No.110, Sec. 1, Jianguo N. Rd., South Dist., Taichung City 40201, Taiwan; missyuping@gmail.com (Y.P.H.)
3 Institute of Medicine, School of Medicine, Chung Shan Medical University, No.110, Sec. 1, Jianguo N. Rd., South Dist., Taichung City 40201, Taiwan
4 Department of Mechanical Engineering, National Chung Cheng University, 168, University Rd., Min Hsiung, Chia Yi 62102, Taiwan; d09420003@ccu.edu.tw
5 Department of Aeronautical Engineering, Vel Tech Rangarajan Dr.Sagunthala R&D Institute of Science and Technology, Avadi, Chennai, India; chaudharyranch@gmail.com
6 Department of Plastic Surgery, Kaohsiung Armed Forces General Hospital, 2, Zhongzheng 1st.Rd., Lingya District, Kaohsiung City 80284, Taiwan
7 Department of Medical Research, Dalin Tzu Chi General Hospital, No. 2, Min-Sheng Rd., Dalin Town, Chiayi 62247, Taiwan
8 Director of Technology Development, Hitspectra Intelligent Technology Co., Ltd., Kaohsiung 80661, Taiwan
* Correspondence: sschin522@gmail.com (S.-C.H.); hcwang@ccu.edu.tw (H.-C.W.)

Abstract: This article provides the supplementary information for the article, "A Systematic Meta-Analysis of Computer-Aided Detection Methods using Hyperspectral Imaging Engineering to Detect Skin Cancer"

Keywords: Skin Cancer, Hyperspectral Imaging Engineering, Meta-Analysis, Melanoma.

Citation: Huang, H.-Y.; Hsiao, Y.-P.; Karmakar, R.; Mukundan, A.; Chaudhary, P.; Hsieh, S.-C.; Wang, H.-C. A Review of Recent Advances in Computer-Aided Detection Methods Using Hyperspectral Imaging Engineering to Detect Skin Cancer. *Cancers* **2023**, *15*, 5634. https://doi.org/10.3390/cancers15235634

Academic Editor: Farrukh Aqil

Received: 25 August 2023
Revised: 20 November 2023
Accepted: 24 November 2023
Published: 29 November 2023

Copyright: © 2023 by the authors. Licensee MDPI, Basel, Switzerland. This article is an open access article distributed under the terms and conditions of the Creative Commons Attribution (CC BY) license (https://creativecommons.org/licenses/by/4.0/).

Materials

Materials includes supporting information for the main article including details about literature search, inclusion and exclusion criteria, primary outcomes, data extraction, additional analyses, study inclusion, quality analysis, QUADAS-2, forest plot, and funnel plot.

A. Literature Search

Articles used in this study were independently searched by authors on the web, specifically, in Google Scholar search engine. Author selected the relevant and recent research paper between 2017-2022 for this review, and duplications were removed. Understanding the abstracts as well as the titles of the identified articles was efficient to avoid the inclusion of articles irrelevant to the purpose of this review. Furthermore, reading the full text facilitated deciding whether the articles met the criteria for inclusion.

B. Inclusion Criteria

This review highlights on studies meeting the mentioned inclusion criteria:
(i) studies with well-defined numerical results such as dataset, sensitivity, accu racy, precision, wavelength, and area under curve
(ii) based on hyperspectral imaging dealing with skin cancer detection

(iii) must be published in the last five years
(iv) publication journal must have an H-index of greater than 50, impact factor greater than 3 and must be in the first quartile (Q1)
(v) studies that have a prospective or retrospective design.
(vi) studies written in English

C. Exclusion Criteria

This review disregards studies falling under the exclusion criteria:
(i) studies with insufficient data
(ii) studies under narrative, systematic review, and meta-analyses
(iii) comments, proceedings, or study protocols
(iv) conference papers

D. Primary Outcomes, Data Extraction, and Additional Analyses

The extraction and cross-checking of the data were done by two authors (Pramod and Arvind). The primary means of communication for data inquiries and validation was email. The process of synthesizing each study was generated by a diagnostic test accuracy and a systematic review consequentially. Data gathered in the meta-analyses were mostly about specificity, sensitivity, and accuracy of the diagnostic performance established on the skin cancer detection in each study.

Furthermore, the subgroup analysis composed of the origin of data was recorded geographically. The skin lesions type, computer-aided diagnosis (CAD) method type, and skin cancer type were also provided in the subgroup table for further analysis.

E. Study Inclusion

A combination of 1,700 results was distinguished when searched thoroughly "Hyperspectral Imaging: skin cancer detection" in Google Scholar. A total of 680 articles were excluded after considering the years they were published because this review solely focuses on articles published in recent years (2017– 2022). Articles with full-text access were also observed making up about 563 articles considered for exclusion. A total of 457 articles were left to be reviewed. Among these 457 records were articles with incomplete data, narrative reviews and meta-analyses, comments, and conference papers that were part of the exclusion criteria. Eventually, ten studies were included in this work. Supplementary Figure 1 shows the selection flowchart.

F. Quality Analysis

A precise, detailed information from a study to be reviewed is essential and considered a good quality for a precise inference. This precise conclusion improves the CAD methods in data training and learning. In this study, a couple of skin lesion images and involved patients with skin cancer are the main contributors of the data needed for training. Furthermore, concerns regarding applicability and risk of bias must be used because not all studies in this review provided a detailed description of the patient enrollment standard, reference standard, and index test. All studies were "low risk" in applicability concern except a study by Hosking et al., were labelled as "unclear risk" because the patient selection is not directly used in this research for meta-analysis. The study by Vinokurov et al., and Zherdeva et al., were labelled as "unclear risk" in terms of patient selection because the skin cancer patients are not selected based on the bias of risk and their number were also not mentioned in research paper. The study by Christensen et al., and Rasanen et al., were labelled as "unclear risk" in terms of index test due to the absence of primary outcomes as well as values for specificity and sensitivity. The study by Christensen et al., Rasanen et al., and Zherdeva et al., also marked as "unclear risk" in flow, timing, and reference standard because of the lack of supporting data for their stated average consistency in diagnostic accuracy. Supplementary Figure 2 shows the quality analysis of ten studies.

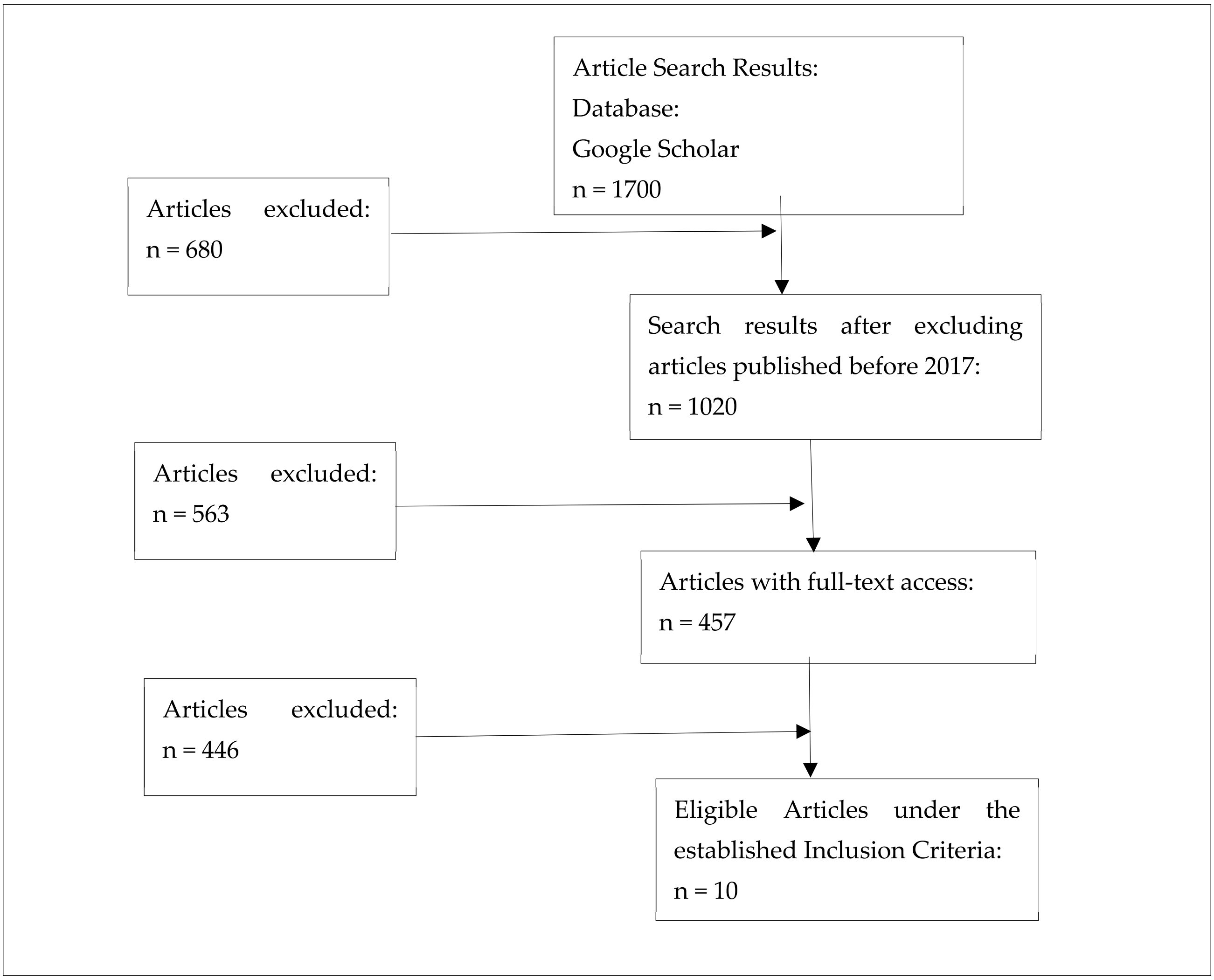

Figure S1. Search Process Flowchart

G. QUADAS-2

This section summarizes the QUADAS-2 outcome of the eight studies for this review. It contains the applicability concerns and the level of risk of bias of the studies based on flow and timing, patient selection, reference standard, and index test. Each study was reviewed under flow and timing, patient selection, reference standard, and index test for the risk of bias as well as under patient selection, reference standard, and index test for applicability concerns. Supplementary Figure 3 shows the graphical result of Quadas-2 domain.

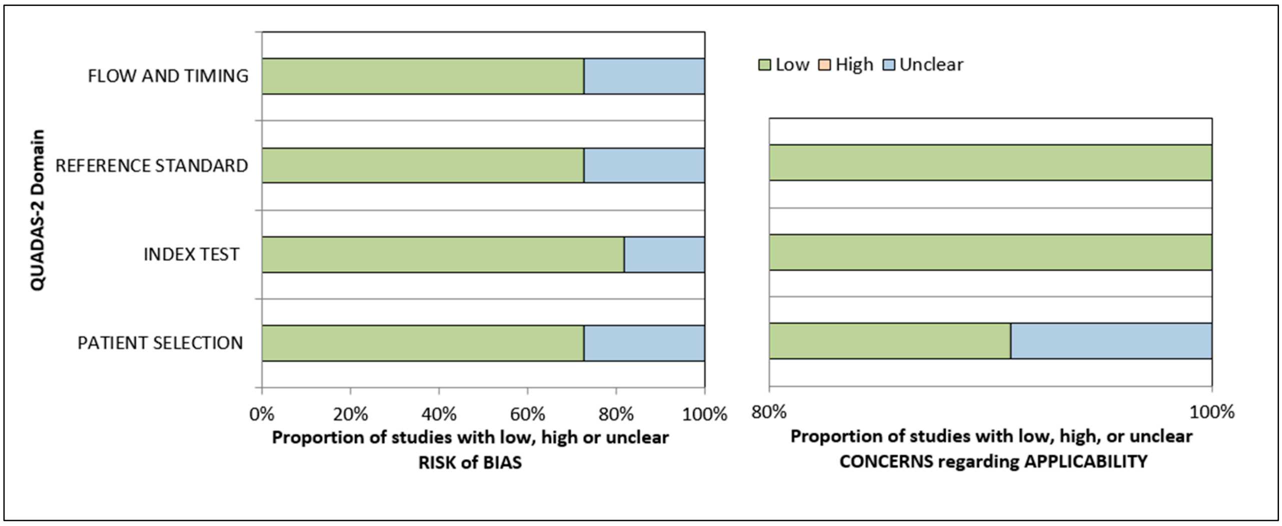

Figure S2. Result summary of QUADAS-2 domain

H. Forest Plot

This section presents forest plots of sensitivity and specificity for different classifications associated in this study such as for the studies involved and for the CAD method. The forest plot explains the quality of the data involved in each classification under the 95% level of confidence with their calculated upper limits and lower limits. The quality of data from forest plots were interpreted in respect with the line of no effect. The line of no effect used in this study was calculated by deriving the average of the sensitivity and specificity of the data involved in each classification. Forest plots reveal the data overlapping the line of no effect describes the data as a low performance data and the data without overlapping the line of no effect shows the high performance of the data. CNN and SKL lie right from line of no action so these methods have significant values for grouped meta-analysis whereas SVM and DI coincide with line of no action so these methods have less significant values in specificity forest plot. It means p-value of CNN and SKL are less than 0.005 CI whereas p-value of SVM and DI are greater than 0.005 CI and results are heterogeneous. Similarly, In sensitivity forest plot, CNN and SVM method have p-value less than 0.005 CI that means these methods are significant. SKL and DI method have p-values greater than 0.005 CI that means these methods are less significant.

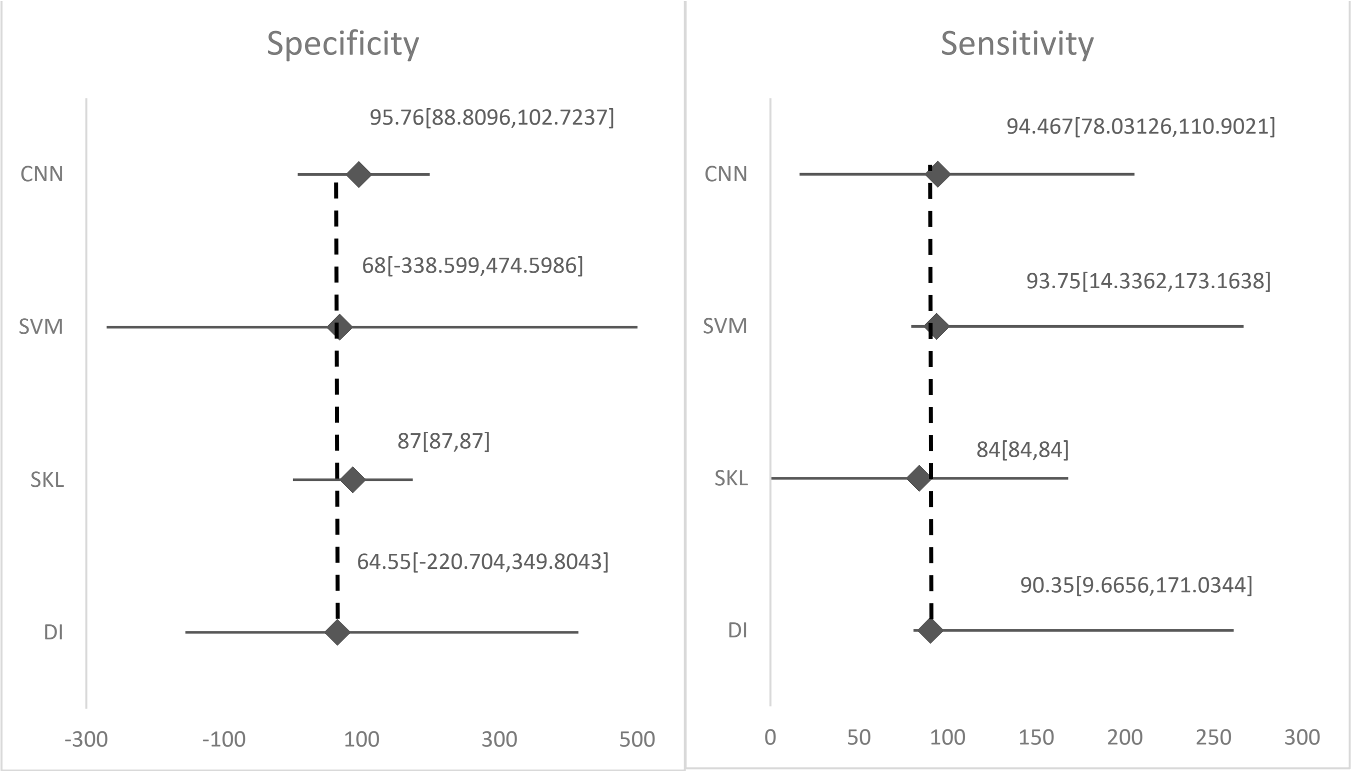

a) b)

Figure S3. Forest plot of sensitivity and specificity (Based on CAD method) (a, b)

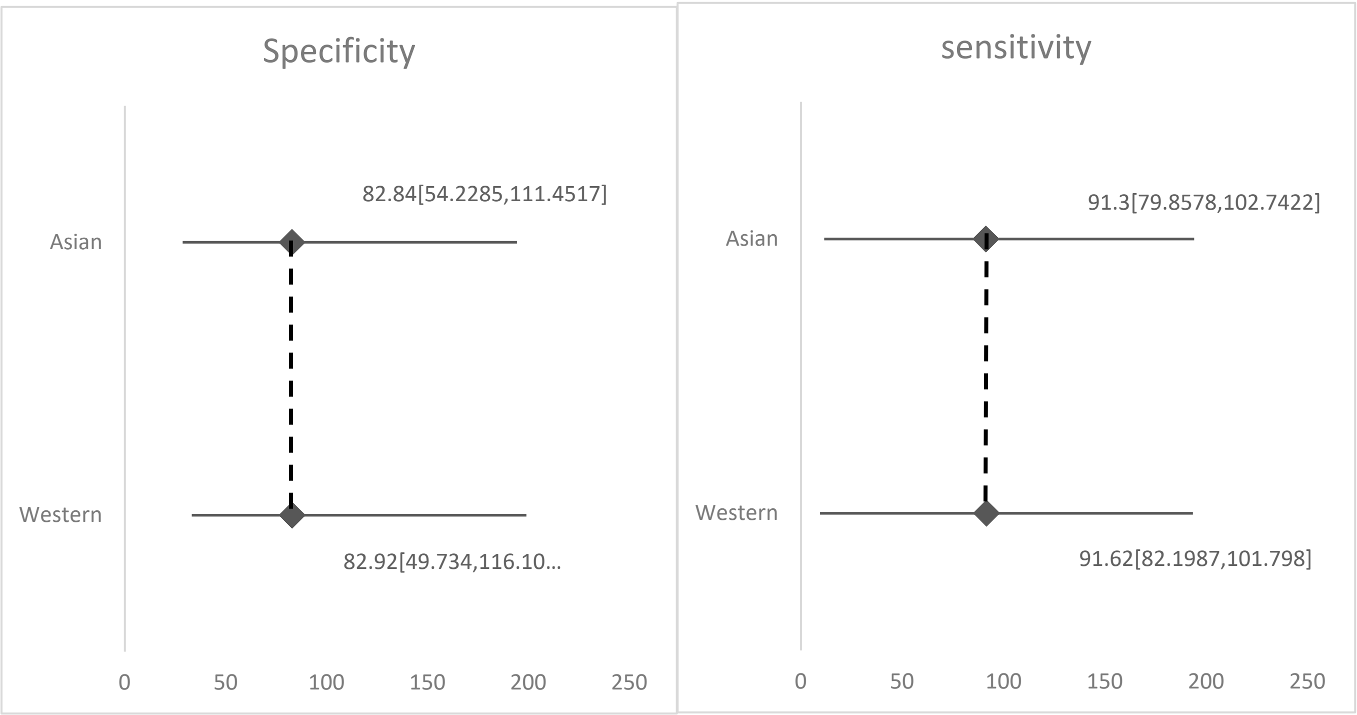

c) d)

Figure S4. Forest plot of sensitivity and specificity (Based on Nationality) (c, d)

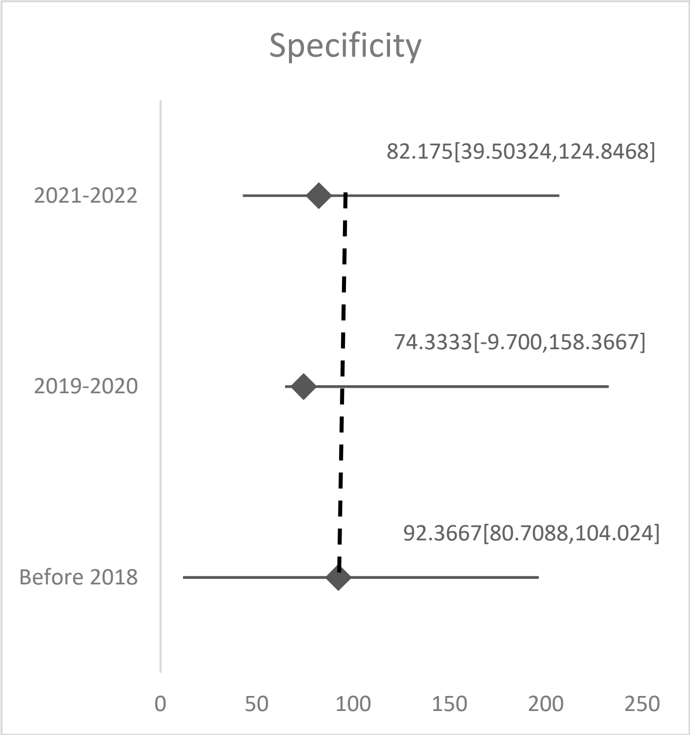

e)

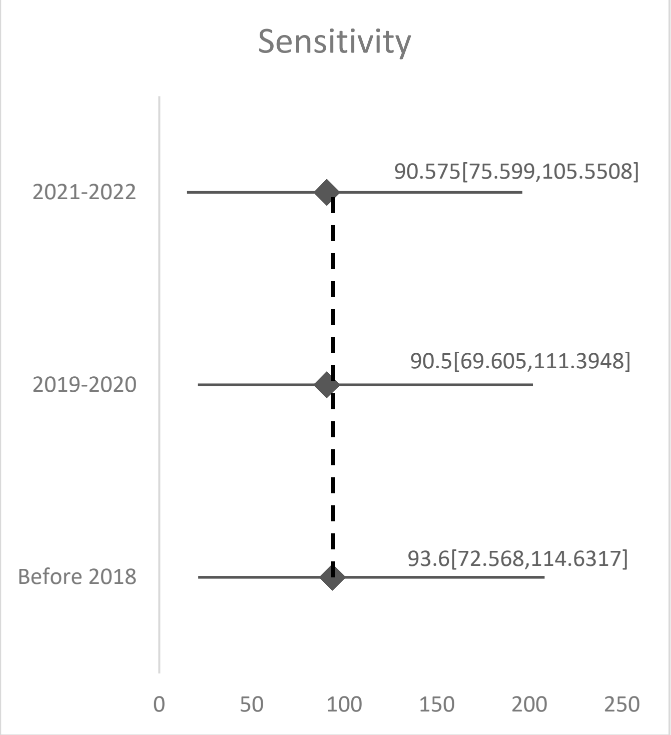

f)

Figure S5. Forest plot of sensitivity and specificity (Based on Year of Publication) (e, f)

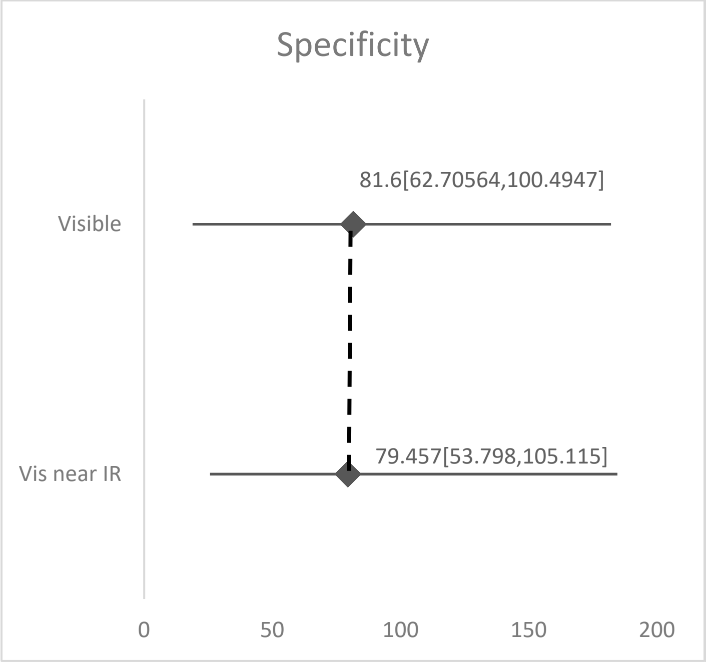

g)

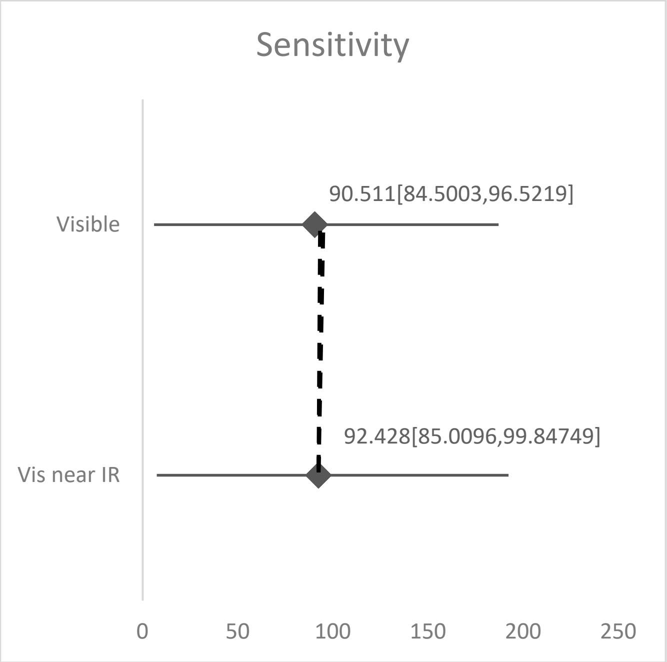

h)

Figure S6. Forest plot of sensitivity and specificity (Based on Band Region) (g, h)

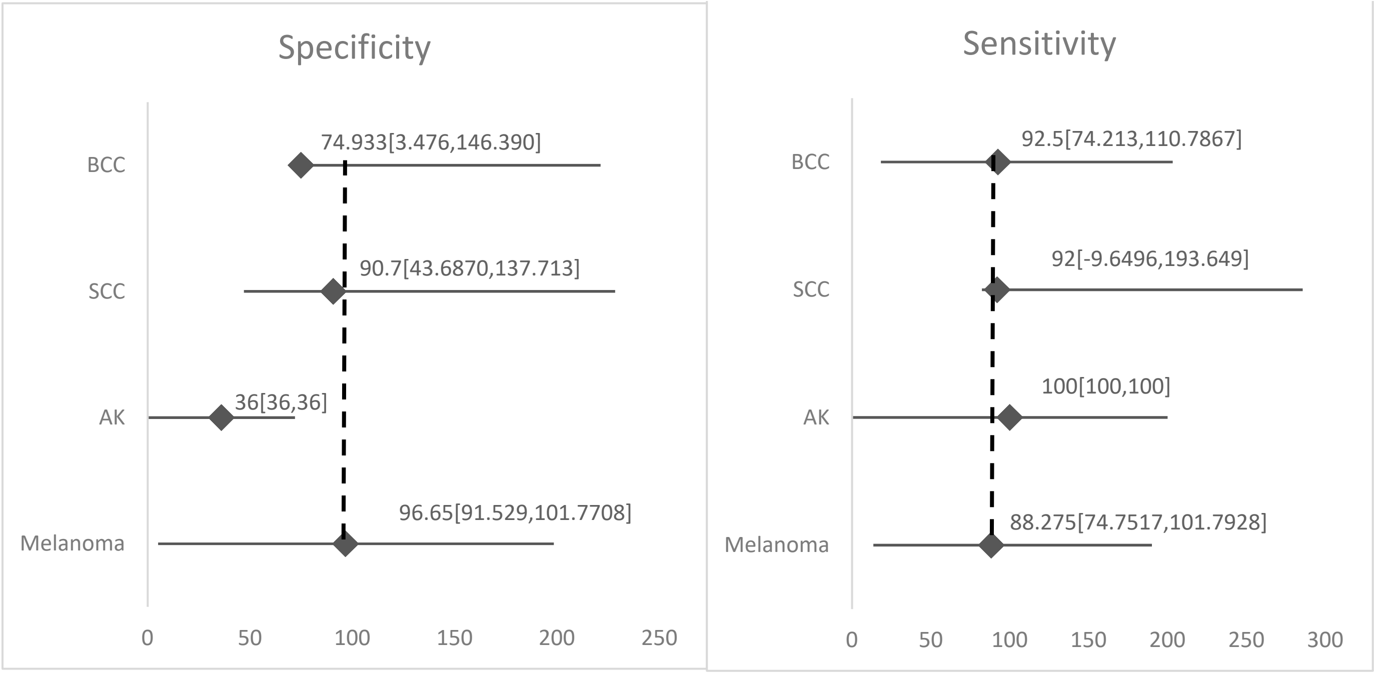

i) j)

Figure S7. Forest plot of sensitivity and specificity (Based on Type of skin cancers) (i, j)

I. Deek`s Funnel Plot

This portion presents the Deek`s funnel plot for different classifications such as nationality, type of CAD method, year of publications, type of skin cancer and wavelength band region. This funnel plot is constructed for evaluating publication bias. It involves the square root of the dataset in y-axis and the odds diagnostic ratio in x-axis. The regression line is constructed to ensure the consideration of the meta-analytical estimate of publication biases. Deek`s funnel plot is the plot of the variability of the individual studies (standard error) against the mean effect size. The results plotted in funnel plot are unsymmetrical that means the studies are selected based on the bias of the mean regression line showing the standard error against odd ratio. From funnel plot, it inferred that the publication bias 2021-2022 and 2019-2020 have high standard error values and high odd ratio. Publication bias before 2018 has low standard error values and odd ratio which are less heterogeneous with p-value of 0.47567 which is more than 0.005 so that these results are more heterogeneous having standard error 3.487521 Similarly, Vis near IR band region has low standard error values with high odd ratio. Visible band region has high standard error values but low diagnostic odd ratio having regression line 1.02613.

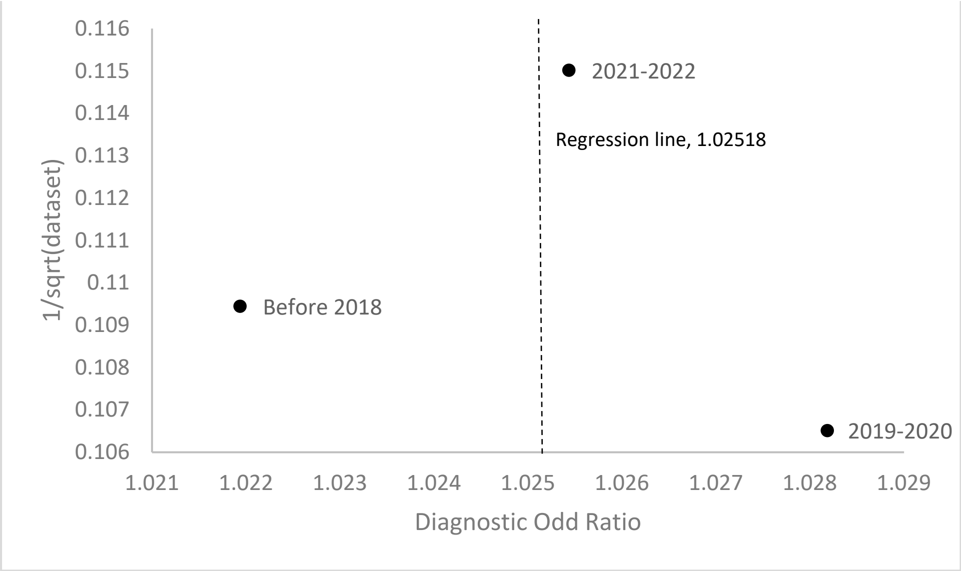

Figure S8. Deek`s funnel plot (year of publication)

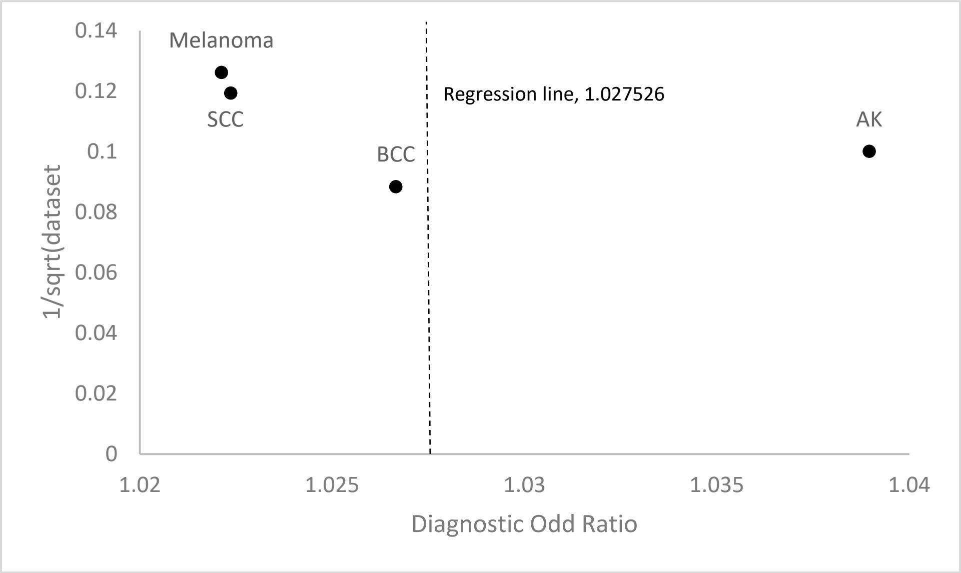

Figure S9. Deek`s funnel plot (type of skin cancer)

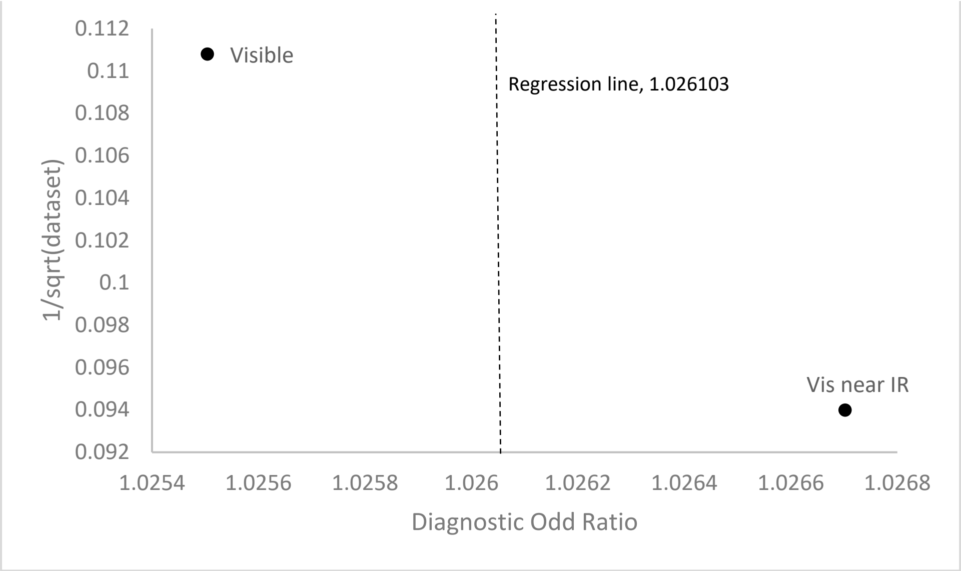

Figure S10. Deek`s funnel plot (wavelength band region)

Materials B

Materials B contains the summary of the calculations as supporting information for the graphs and figures generated and explained in the main article. Materials B includes the summary of computations for forest plot and Deek`s funnel plot.

A. Summary of Computations for Forest Plots

This section contains the computations obtained for each forest plot. It contains the mean and confidence level that are essential in plotting the forest plots.

Sensitivity (CNN)			*Specificity(CNN)*	
Mean	94.46667		Mean	95.76666667
Standard Error	3.819831		Standard Error	1.616924378
Median	96.8		Median	95.7
Standard Deviation	6.616142		Standard Deviation	2.800595175
Sample Variance	43.77333		Sample Variance	7.843333333
Skewness	-1.38964		Skewness	0.107059388
Range	12.6		Range	5.6
Minimum	87		Minimum	93
Maximum	99.6		Maximum	98.6
Sum	283.4		Sum	287.3
Count	3		Count	3
Confidence Level(95.0%)	16.43541		Confidence Level(95.0%)	6.957064089
Upper CI	110.9021		Upper CI	102.7237308
Lower CI	78.03126		Lower CI	88.80960258

Sensitivity(SVM)	
Mean	93.75
Standard Error	6.25
Median	93.75
Standard Deviation	8.838835
Sample Variance	78.125
Range	12.5
Minimum	87.5
Maximum	100
Sum	187.5
Count	2
Confidence Level(95.0%)	79.41378
Upper CI	173.1638
Lower CI	14.33622

Sensitivity(SVM)	
Mean	93.75
Standard Error	6.25
Median	93.75
Standard Deviation	8.838834765
Sample Variance	78.125
Range	12.5
Minimum	87.5
Maximum	100
Sum	187.5
Count	2
Confidence Level(95.0%)	79.4137796
Upper CI	173.1637796
Lower CI	14.3362204

Sensitivity(SKL)	
Mean	84
Standard Error	0
Median	84
Range	0
Minimum	84
Maximum	84
Sum	84
Count	1
Confidence Level(95.0%)	N/A
Upper CI	84
Lower CI	84

Specificity(SKL)	
Mean	87
Standard Error	0
Median	87
Range	0
Minimum	87
Maximum	87
Sum	87
Count	1
Confidence Level(95.0%)	N/A
Upper CI	87
Lower CI	87

Sensitivity (DI)	
Mean	90.35
Standard Error	6.35
Median	90.35
Standard Deviation	8.980256
Sample Variance	80.645
Range	12.7
Minimum	84
Maximum	96.7
Sum	180.7
Count	2
Confidence Level(95.0%)	80.6844
Upper CI	171.0344
Lower CI	9.6656

Specificity (DI)	
Mean	64.55
Standard Error	22.45
Median	64.55
Standard Deviation	31.749094
Sample Variance	1008.005
Range	44.9
Minimum	42.1
Maximum	87
Sum	129.1
Count	2
Confidence Level(95.0%)	285.2543
Upper CI	349.8043
Lower CI	-220.7043

Table S1. SENSITIVITY AND SPECIFICITY FOR FOREST PLOT (CAD METHOD)

Sensitivity of Asian		*Specificity of Asian*	
Mean	91.3	Mean	82.84
Standard Error	4.121165	Standard Error	10.30517346
Median	96.7	Median	94.4
Standard Deviation	9.215205	Standard Deviation	23.04306837
Sample Variance	84.92	Sample Variance	530.983
Kurtosis	-2.24127	Kurtosis	4.51199136
Skewness	-0.67489	Skewness	-2.113951478
Range	21	Range	53.6
Minimum	79	Minimum	42.1
Maximum	100	Maximum	95.7
Sum	456.5	Sum	414.2
Count	5	Count	5
Confidence Level(95.0%)	11.44219	Confidence Level(95.0%)	28.61174841
Upper CI	102.7422	Upper CI	111.4517484
Lower CI	79.85781	Lower CI	54.22825159

Sensitivity of Western		*Sensitivity of Western*	
Mean	91.62	Mean	91.62
Standard Error	3.393287	Standard Error	3.393287492
Median	87.5	Median	87.5
Standard Deviation	7.587621	Standard Deviation	7.587621498
Sample Variance	57.572	Sample Variance	57.572
Kurtosis	-3.05894	Kurtosis	-3.05894221
Skewness	0.463232	Skewness	0.463231909
Range	16	Range	16
Minimum	84	Minimum	84
Maximum	100	Maximum	100
Sum	458.1	Sum	458.1
Count	5	Count	5
Confidence Level(95.0%)	9.421276	Confidence Level(95.(	9.421276446
Upper CI	101.0413	Upper CI	101.0412764
Lower CI	82.19872	Lower CI	82.19872355

Table S2. SENSITIVITY AND SPECIFICITY FOR FOREST PLOT (NATIONALITY)

Sensitivity (2021-2022)		*Specificity (2021-2022)*	
Mean	90.575	Mean	82.175
Standard Error	4.705737	Standard Error	13.408478
Median	91.85	Median	94
Standard Deviation	9.411473	Standard Deviation	26.816957
Sample Variance	88.57583	Sample Variance	719.14917
Kurtosis	-2.38739	Kurtosis	3.855944
Skewness	-0.50513	Skewness	-1.954902
Range	20.6	Range	56.5
Minimum	79	Minimum	42.1
Maximum	99.6	Maximum	98.6
Sum	362.3	Sum	328.7
Count	4	Count	4
Confidence Level(95.0%)	14.97575	Confidence Level(95.0%)	42.671762
Upper CI	105.5508	Upper CI	124.84676
Lower CI	75.59925	Lower CI	39.503238

Sensitivity (2019-2020)		*Sensitivity (2019-2020)*	
Mean	90.5	Mean	90.5
Standard Error	4.856267	Standard Error	4.8562674
Median	87.5	Median	87.5
Standard Deviation	8.411302	Standard Deviation	8.4113019
Sample Variance	70.75	Sample Variance	70.75
Skewness	1.400816	Skewness	1.4008159
Range	16	Range	16
Minimum	84	Minimum	84
Maximum	100	Maximum	100
Sum	271.5	Sum	271.5
Count	3	Count	3
Confidence Level(95.0%)	20.89483	Confidence Level(95.0%)	20.894832
Upper CI	111.3948	Upper CI	111.39483
Lower CI	69.60517	Lower CI	69.605168

Sensitivity (Before 2018)			Specificity (Before 2018)	
Mean	93.6		Mean	92.36667
Standard Error	4.888081		Standard Error	2.709448
Median	96.8		Median	94.4
Standard Deviation	8.466404		Standard Deviation	4.692902
Sample Variance	71.68		Sample Variance	22.02333
Skewness	-1.45786		Skewness	-1.58373
Range	16		Range	8.7
Minimum	84		Minimum	87
Maximum	100		Maximum	95.7
Sum	280.8		Sum	277.1
Count	3		Count	3
Confidence Level(95.0%)	21.03171		Confidence Level(95.0%)	11.65782
Upper CI	114.6317		Upper CI	104.0245
Lower CI	72.56829		Lower CI	80.70885

Table S3. SENSITIVITY AND SPECIFICITY FOR FOREST PLOT (Year of Publication)

Sensitivity (BCC)			Specificity (BCC)	
Mean	92.5		Mean	74.93333
Standard Error	4.250098		Standard Error	16.60766
Median	96.7		Median	87
Standard Deviation	7.361386		Standard Deviation	28.76531
Sample Variance	54.19		Sample Variance	827.4433
Skewness	-1.73169		Skewness	-1.55552
Range	12.8		Range	53.6
Minimum	84		Minimum	42.1
Maximum	96.8		Maximum	95.7
Sum	277.5		Sum	224.8
Count	3		Count	3
Confidence Level(95.0%)	18.2867		Confidence Level(95.0%)	71.457
Upper CI	110.7867		Upper CI	146.3903
Lower CI	74.2133		Lower CI	3.47633

Sensitivity (SCC)	
Mean	92
Standard Error	8
Median	92
Standard Deviation	11.31371
Sample Variance	128
Range	16
Minimum	84
Maximum	100
Sum	184
Count	2
Confidence Level(95.0%)	101.6496
Upper CI	193.6496
Lower CI	-9.64964

Specificity (SCC)	
Mean	90.7
Standard Error	3.7
Median	90.7
Standard Deviation	5.23259018
Sample Variance	27.38
Range	7.4
Minimum	87
Maximum	94.4
Sum	181.4
Count	2
Confidence Level(95.(	47.0129575
Upper CI	137.712958
Lower CI	43.6870425

Sensitivity (AK)	
Mean	100
Standard Error	0
Median	100
Range	0
Minimum	100
Maximum	100
Sum	100
Count	1
Confidence Level(95.0%)	N/A
Upper CI	100
Lower CI	100

Specificity (AK)	
Mean	36
Standard Error	0
Median	36
Range	0
Minimum	36
Maximum	36
Sum	36
Count	1
Confidence Level(95.0%)	N/A
Upper CI	36
Lower CI	36

Sensitivity (melanoma)			Specificity (melanoma)	
Mean	88.275		Mean	96.65
Standard Error	4.247622		Standard Error	1.609088769
Median	87.25		Median	96.8
Standard Deviation	8.495244		Standard Deviation	3.218177538
Sample Variance	72.16917		Sample Variance	10.35666667
Kurtosis	1.765705		Kurtosis	-3.390519181
Skewness	0.709047		Skewness	-0.162198205
Range	20.6		Range	7
Minimum	79		Minimum	93
Maximum	99.6		Maximum	100
Sum	353.1		Sum	386.6
Count	4		Count	4
Confidence Level(95.0%)	13.51783		Confidence Level(95.0%)	5.120838608
Upper CI	101.7928		Upper CI	101.7708386
Lower CI	74.75717		Lower CI	91.52916139

Table S4. SENSITIVITY AND SPECIFICITY FOR FOREST PLOT (TYPE OF SKIN CANCER)

Sensitivity (Visible band)			Specificity (Visible band)	
Mean	90.51111		Mean	81.6
Standard Error	2.606586		Standard Error	8.19368
Median	87.5		Median	93
Mode	84		Mode	87
Standard Deviation	7.819758		Standard Deviation	24.58104
Sample Variance	61.14861		Sample Variance	604.2275
Kurtosis	-1.75264		Kurtosis	0.598482
Skewness	-0.01915		Skewness	-1.4965
Range	21		Range	64
Minimum	79		Minimum	36
Maximum	100		Maximum	100
Sum	814.6		Sum	734.4
Count	9		Count	9
Confidence Level(95.0%)	6.010798		Confidence Level(95.0%)	18.89466
Upper CI	96.52191		Upper CI	100.4947
Lower CI	84.50031		Lower CI	62.70534

Sensitivity (Vis near IR)			Specificity (Vis near IR)	
Mean	92.42857		Mean	79.457143
Standard Error	3.03195		Standard Error	10.486042
Median	96.7		Median	94.4
Standard Deviation	8.021786		Standard Deviation	27.743459
Sample Variance	64.34905		Sample Variance	769.69952
Kurtosis	-0.75149		Kurtosis	-0.7496551
Skewness	-0.75564		Skewness	-1.2202437
Range	21		Range	64
Minimum	79		Minimum	36
Maximum	100		Maximum	100
Sum	647		Sum	556.2
Count	7		Count	7
Confidence Level(95.0%)	7.418915		Confidence Level(95.0%)	25.65842
Upper CI	99.84749		Upper CI	105.11556
Lower CI	85.00966		Lower CI	53.798723

Table S5. SENSITIVITY AND SPECIFICITY FOR FOREST PLOT (BAND REGION)

B. *Summary of Computations for Deek`s Funnel Plots*

This section shows the computations obtained for each Deek`s funnel plot. It contains the regression statistics and the number of observations needed in the funnel plot.

Regression Statistics	
Multiple R	1
R Square	1
Adjusted R Square	65535
Standard Error	0
Observations	2

Table S6. REGRESSION STATISTICS (NATIONALITY)

Regression Statistics	
Multiple R	0.1691629
R Square	0.0286161
Adjusted R Squar	-0.457076
Standard Error	0.0178412
Observations	4

Table S7. REGRESSION STATISTICS (CAD METHOD)

Regression Statistics	
Multiple R	0.273601
R Square	0.074858
Adjusted R Square	-0.85028
Standard Error	0.005876
Observations	3

Table S8. REGRESSION STATISTICS (YEAR OF PUBLICATION)

Regression Statistics	
Multiple R	0.5592617
R Square	0.3127737
Adjusted R Square	-0.0308395
Standard Error	0.0176322
Observations	4

Table S9. REGRESSION STATISTICS (TYPE OF SKIN CANCER)

Regression Statistics	
Multiple R	1
R Square	1
Adjusted R Squar	65535
Standard Error	0
Observations	2

Table S10. REGRESSION STATISTICS (BAND REGION)

Materials C

Materials C shows the computations of ANOVA information for both forest plots and Deek`s funnel plots with numerical results of ANOVA analysis for construction of graphs.

A. Summary of Computations for Forest Plots

This section shows the numerical results of ANOVA analysis in which it is showing the values of regression line along with residual values for each study carried out in forest plot. This computation also shows the p-value of forest plot is 0.241231.

Groups	*Count*	*Sum*	*Average*	*Variance*		
Column 1	11	1006.06	91.46	57.0224		
Column 2	11	911.68	82.88	498.1276		
ANOVA						
Source of Variation	*SS*	*df*	*MS*	*F*	*P-value*	*F crit*
Between Groups	404.8902	1	404.8902	1.45867	0.241231	4.351244
Within Groups	5551.5	20	277.575			
Total	5956.39	21				

Table S11. ANOVA ANALYSIS OF FOREST PLOT

B. Deek`s Funnel Plot Computation of p value

This section shows the computations obtained for the p-value needed in the Deek`s funnel plot. A p-value is essential in determining the heterogeneity of the data involved. It shows p-values below 0.05 suggest heterogeneity. By contrast, p-values above 0.05 suggest no heterogeneity.

	df	*SS*	*MS*				
Regression	1	0.000332753	0.0003328				
Residual	0	0	65535				
Total	1	0.000332753					

	Coefficients	*Standard Error*	*t Stat*	*Lower 95%*	*Upper 95%*	*Lower 95.0%*	*Upper 95.0%*
Intercept	-40.845123	0	65535	-40.845123	-40.84512296	-40.84512296	-40.84512296
X Variable 1	39.95879802	0	65535	39.95879802	39.95879802	39.95879802	39.95879802

Table S12. ANOVA ANALYSIS (NATIONALITY)

	df	*SS*	*MS*	*F*	*Significance F*			
Regression	1	1.87542E-05	1.87542E-05	0.058918211	0.830837059			
Residual	2	0.000636619	0.000318309					
Total	3	0.000655373						

	Coefficients	*Standard Error*	*t Stat*	*P-value*	*Lower 95%*	*Upper 95%*	*Lower 95.0%*	*Upper 95.0%*
Intercept	1.179429021	4.395530949	0.268324586	0.813591465	-17.73301422	20.09187226	-17.73301422	20.09187226
X Variable 1	-1.039409218	4.28214914	-0.242730737	0.830837059	-19.4640099	17.38519147	-19.4640099	17.38519147

Table S13. ANOVA ANALYSIS (CAD METHODS)

	df	*SS*	*MS*	*F*	*Significance F*			
Regression	1	2.79374E-06	2.79374E-06	0.08091488	0.823570122			
Residual	1	3.45269E-05	3.45269E-05					
Total	2	3.73206E-05						

	Coefficients	*Standard Error*	*t Stat*	*P-value*	*Lower 95%*	*Upper 95%*	*Lower 95.0%*	*Upper 95.0%*
Intercept	0.497315173	1.360467637	0.365547228	0.77689172	-16.7890652	17.7836955	-16.78906516	17.7836955
X Variable 1	-0.37748374	1.327040132	-0.284455408	0.82357012	-17.2391274	16.48415987	-17.23912735	16.48415987

Table S14. ANOVA ANALYSIS (YEAR OF PUBLICATION)

	df	*SS*	*MS*	*F*	*Significance F*			
Regression	1	0.000282993	0.000282993	0.91024944	0.440738268			
Residual	2	0.000621792	0.000310896					
Total	3	0.000904785						

	Coefficients	*Standard Error*	*t Stat*	*P-value*	*Lower 95%*	*Upper 95%*	*Lower 95.0%*	*Upper 95.0%*
Intercept	1.371279063	1.32367951	1.035960029	0.40905555	-4.324054194	7.06661232	-4.324054194	7.06661232
X Variable 1	-1.22902528	1.288192021	-0.954069935	0.44073827	-6.771668193	4.313617638	-6.771668193	4.313617638

Table S15. ANOVA ANALYSIS (TYPE OF SKIN CANCER)

	df	*SS*	*MS*				
Regression	1	0.000141204	0.000141204				
Residual	0	0	65535				
Total	1	0.000141204					

	Coefficients	*Standard Error*	*t Stat*	*Lower 95%*	*Upper 95%*	*Lower 95.0%*	*Upper 95.0%*
Intercept	14.49609744	0	65535	14.49609744	14.49609744	14.49609744	14.49609744
X Variable 1	-14.02754591	0	65535	-14.02754591	-14.02754591	-14.02754591	-14.0275459

Table S16. ANOVA ANALYSIS (WAVELENGTH BAND REGION)
